# Preliminary Ecological Data on the Cocco's Lanternfish *Lobianchia gemellarii* (Cocco, 1838) (Osteichthyes: Myctophidae) in Mediterranean With New Records in the Northern Ionian Sea

**DOI:** 10.1002/ece3.70731

**Published:** 2024-12-23

**Authors:** Cristina Turco, Letizia Sion, Gabriele Galasso, Gianfranco D'Onghia, Francesca Capezzuto

**Affiliations:** ^1^ Department of Biosciences, Biotechnologies and Environment University of Bari Aldo Moro, CoNISMa LRU of Bari University Bari Italy

**Keywords:** ecological data, first record, lantern fish, Mediterranean Sea, Myctophidae, stomach content

## Abstract

This paper presents preliminary ecological data on the Cocco's lanternfish *Lobianchia gemellarii* (Cocco, 1838) (Osteichthyes: Myctophidae) in the Central Mediterranean and its first records from the northern Ionian Sea. A total of 28 specimens of lantern fish were collected using an experimental trawl net between August and September 2023 in a depth range of 500–701 m in the northern Ionian Sea (central Mediterranean), as part of the MEDITS project. Their morphological traits, the presence and arrangement of photophores together with the otolith characteristics allowed the identification of these specimens as *L. gemellarii* . In the study area, the standard length of individuals was up to a maximum of 109 mm, which is the largest size ever found for the species. A length‐age key was estimated through otoliths reading. The stomach content analysis for all individuals is also reported. Plastic fibers were found in the gut contents of two individuals. This study not only represents the first record of the species in the northern Ionian Sea but also provides information and a new contribution to the knowledge on the role of the species in the Mediterranean marine food web.

## Graphical abstract

This paper presents preliminary ecological data on the Cocco's lanternfish *Lobianchia gemellarii* (Cocco, 1838) (Osteichthyes: Myctophidae) in the Central Mediterranean and its first records from the northern Ionian Sea. This study also provides information and a new contribution to the knowledge on the role of the species in the Mediterranean marine food web.

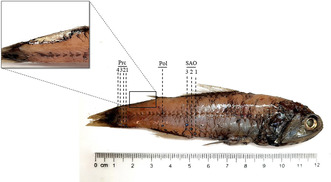

## 1 Introduction

Mesopelagic fish occupy the upper 1000 m of the oceans and are ubiquitous in the world, with high diversity and abundance, forming deep scattering layers (DSLs) detected by echosounders (Catul, Gauns, and Karuppasamy 2011). Research on their ecology has intensified in recent years and has focused on life history, morphology, behavior, trophodynamics, and biomass distribution (Loots, Koubbi, and Duhamel 2007; Bernal et al. 2015; Freer et al. 2019; Clavel‐Henry et al. 2020). However, many gaps still remain.

The deeper or deepest DSL are inhabited by a mix of mesopelagic species, mainly myctophids (Kapelonis et al. 2023). The family Myctophidae, order Myctophiformes, consists of small mesopelagic fish that play a key role in the energy transfer from deep environments to higher trophic levels due to their extensive migrations throughout the water column (Kozlov 1995), providing a significant source of energy to many predators (Mann 1984; Davison et al. 2013). In the marine food web, they occupy an intermediate trophic position (Cherel et al. 2010), preying on mainly pelagic crustaceans and mesozooplankton (Hopkins and Baird 1977; Hopkins and Gartner 1992; Pakhomov et al. 1996; Pusch et al. 2004; Brodeur and Yamamura 2005) and constituting an important part of the diet of diverse predators, such as large fish, squids, marine birds, and marine mammals (Pauly et al. 1998; Williams et al. 2001; Hunt et al. 2005; Connan, Cherel, and Mayzaud 2007; Bernal, Olivar, and Fernández de Puelles 2013).

Studies on the feeding behaviors of the most abundant myctophids have spanned various areas of the world's oceans, engaging numerous researchers over the years (e.g., Hopkins and Baird 1977, 1985; Rowedder 1979; Clarke 1980; Sameoto 1988; Furuhashi and Shimazaki 1989; Hopkins and Gartner 1992; Hopkins, Sutton, and Lancraft 1996; Moku et al. 2000; Watanabe and Kawaguchi 2003; Pusch et al. 2004; Brodeur and Yamamura 2005; Uchikawa, Yamamura, and Hattori 2008; Shreeve et al. 2009; Cherel et al. 2010; Van Noord et al. 2013; Fanelli et al. 2014; Valls et al. 2014). However, the understanding of Mediterranean species' feeding habits remains limited, with attention mainly directed toward *Hygophum benoiti* (Cocco 1838) and *Myctophum punctatum* (Rafinesque, 1810) (Scotto Di Carlo et al. 1982), *Lampanyctus crocodilus* (Risso, 1810) (Stefanescu and Cartes 1992), and *Lampanyctus pusillus* (Johnson, 1890) (Bernal, Olivar, and Fernández de Puelles 2013). Furthermore, significant gaps persist in the knowledge of Mediterranean myctophid ecology, biology, and distribution.

To date, 20 species of myctophids have been recorded in the Mediterranean basin (Hulley 1984; Battaglia et al. 2010, 2015; Olivar et al. 2012; Cavallaro et al. 2016). In particular, the genus *Lobianchia* is represented by two species: *Lobianchia gemellarii* (Cocco 1838) and *Lobianchia dofleini* (Zugmayer, 1911). In this study, we focused on the Cocco's lanternfish *L. gemellarii* , a high‐oceanic mesopelagic species distributed in the Pacific, Indian, and Atlantic oceans and in the Mediterranean and Black Sea (Nafpaktitis et al. 1977; Hulley 1984; Cihangir et al. 2003; Koukouras 2010; Borg et al. 2023). The Cocco's lanternfish has been previously described in the Aegean Sea (Cihangir et al. 2003). Concerning the Ionian Sea, the species has been included in the checklist of marine species from Greece (Koukouras 2010). Previously, the records of this species in the northern Ionian Sea (Maiorano et al. 2010; Capezzuto et al. 2010) were erroneously reported instead of *L. dofleini* due to mistakes in species identification. In fact, the species *L. gemellarii* is often confused with *L. dofleini* (Zugmayer, 1911), which is more abundant and common than Cocco's lanternfish throughout the Mediterranean Sea (Nafpaktitis et al. 1977; Hulley 1984; Fischer, Bauchot, and Schneider 1987).

The aim of this work was to report the first records of *L. gemellarii* in the northern Ionian Sea (central Mediterranean) and update the ecological knowledge of this species in the Mediterranean Sea, reporting the first preliminary data regarding its distribution, length/weight relationship, otolith structure, and feeding habits.

## 2 Materials and Methods

**FIGURE 1:**
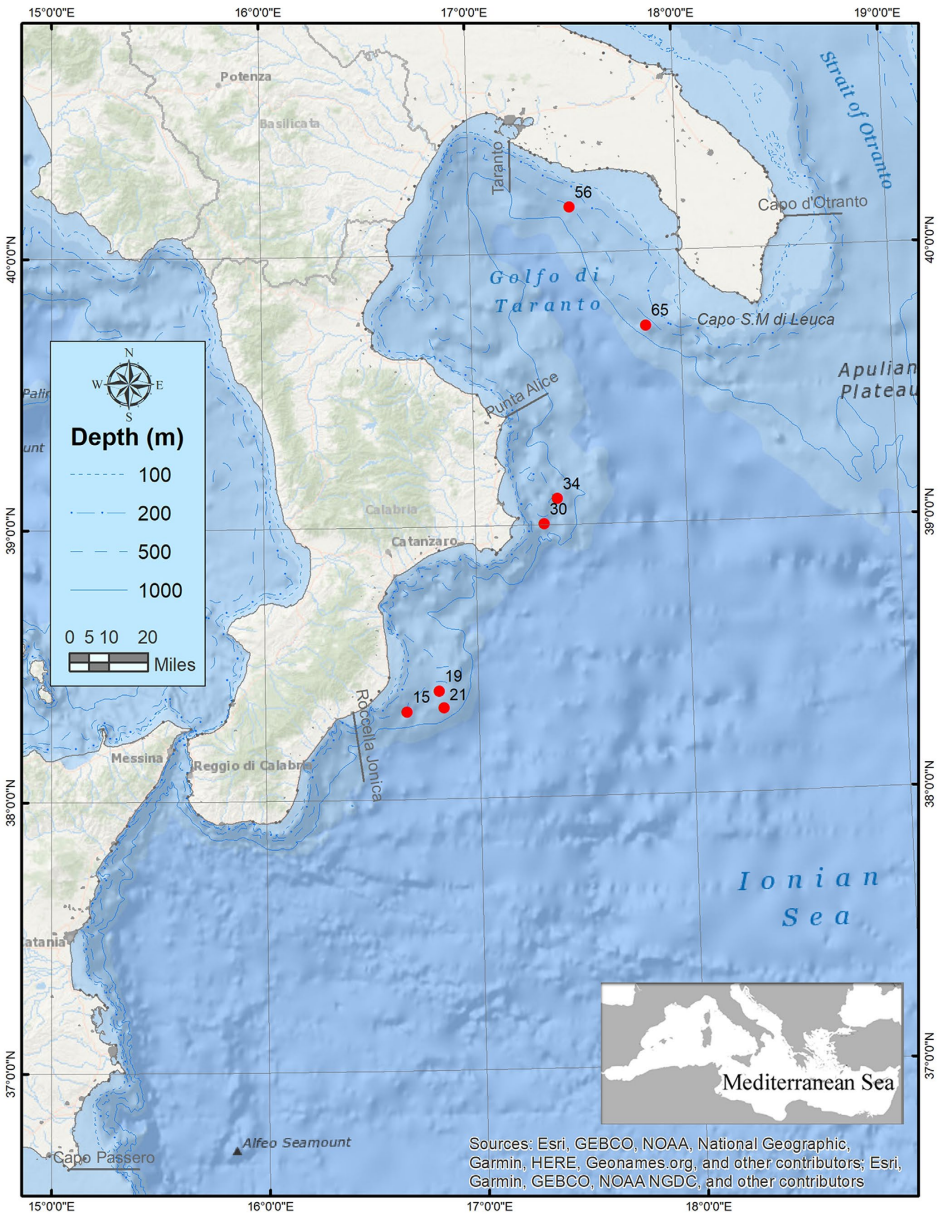
Location of the sampling sites of *L. gemellarii*.

During the framework of the MEDiterranean International Bottom Trawl Survey (MEDITS) (Bertrand et al. 2002) carried out on fishing bottoms from Otranto to Capo Passero (Geographical Sub‐Area 19, GSA 19) between August and September 2023, 28 specimens of the Cocco's lanternfish *Lobianchia gemellarii* were caught. One specimen was sampled at a depth of 660 m off the coast of Riace (38.32250°N, 16.66350°E), four individuals at a depth of 590 m off the coast of Punta Stilo (38.39716°N, 16.81583°E) and 18 individuals at a depth of 701 m in the same area (38.33516°N, 16.83733°E), one sampled at a depth of 647 m off the coast of Capo Colonna (39.00433°N, 17.32666°E), two individuals sampled at a depth of 627 m off the coast of Crotone (39.09633°N, 17.39116°E), one collected at a depth of 500 m off the coast of Maruggio (40.16783°N, 17.48533°E), and lastly one specimen was sampled at a depth of 666 m off the coast of Ugento (39.72516°N, 17.83583°E). All mentioned occurrences are shown in Figure 1.

**FIGURE 2:**
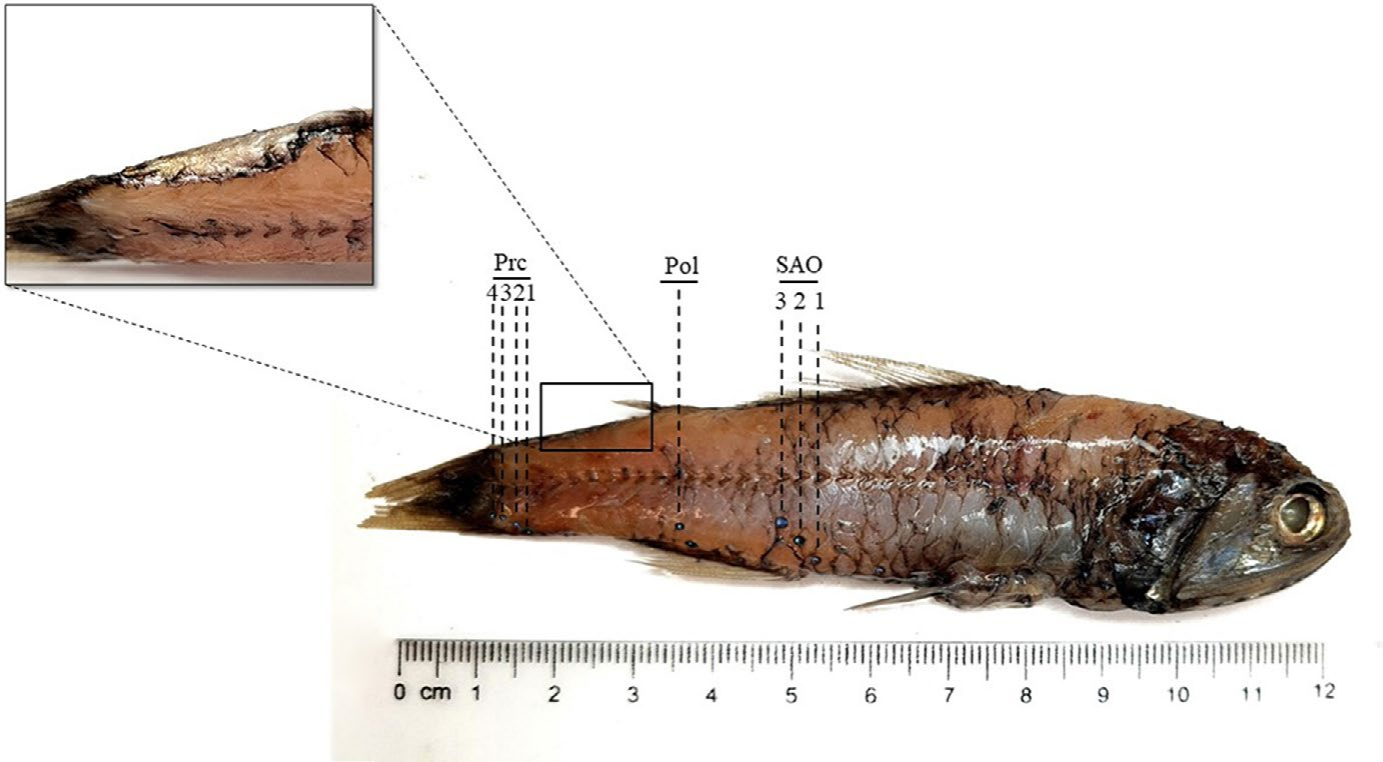
Male specimen of *L. gemellarii* sampled in the northern Ionian Sea. Pol, postero‐lateral photophore; Prc, pre‐caudal photophores; SAO, supra‐anal photophores.

*L. gemellarii* was identified using the diagnostic characters described by Nafpaktitis et al. (1977), Hulley (1984), and Fischer, Bauchot, and Schneider (1987) and from the arrangement of photophores reported by Sutton et al. (2020). The body is slender and compressed in its ventral part, with a large head with a blunt snout and small eyes. The jaws extend beyond the posterior orbital margin and the maxilla is slightly expanded posteriorly. The dorsal fin base is slightly longer than the anal fin base. Regarding the presence and the position of photophores, each individual had: a postero‐lateral photophore (Pol) midway between the lateral line and the anal‐fin base or lower; three supra‐anal photophores (SAO) on a straight or slightly curved line, with concavity directed postero‐ventrally; four evenly spaced precaudal photophores (Prc); the distance between Prc4 and Prc3 was always shorter than the distance between Prc1 and Prc3 (Figure 2).

The identification was supported by otolith shape (Smale, Watson, and Hecht 1995; Lombarte et al. 2006; Schwarzhans 2013; Lin et al. 2017; http://aforo.cmima.csic.es/index.jsp). For each specimen of *L. gemellarii* sampled, the standard length (SL to the nearest 1 mm) and weight (W to the nearest 0.1 g) were recorded using a millimetric ichthyometric table and a digital balance, respectively. The length–frequency distribution (1 mm size classes) was performed and the standard length–body weight relationship was computed according to the power curve function W = *a*SL^*b*^, where *a* is the intercept and *b* the allometric coefficient. Values of *b* significantly different from 3 indicated that growth in weight is relatively faster than that in length (positive allometry *b* > 3) or lower (negative allometry *b* < 3) (Olivar, Moli, and Bernal 2013).

The sex was identified by the presence/absence of the supra‐caudal luminous gland.

The sagittal otoliths of *L. gemellarii* were collected from all specimens, cleaned, levigated, immersed in glycerine, and observed with a stereomicroscope LEICA M165C under reflected light against a dark background. All otoliths showed the ring pattern common to teleost fish, opaque and transparent rings laid down around an opaque *nucleus*, corresponding to fast and slow growth phases (Williams and Bedford 1974). The age and growth estimations were performed by band count, with one opaque band followed by a transparent one assumed to be an *annulus* (Panfili et al. 2002).

Some scales were removed from the lateral line and collected, measured, and photographed.

The stomachs were removed from all 28 specimens, stored in 70% ethanol solution, and kept frozen (−20°C) until the time of analysis. The food items in the stomach contents were identified using a LEICA M165C stereomicroscope to the lowest possible taxonomic level and classified using manuals and dichotomous keys.

The frequency and contribution of each prey in the diet of *L. gemellarii* was estimated by calculating the frequency of occurrence (%*F* = number of stomachs containing prey *i*/total number of stomachs containing prey × 100), percentage by number (%*N* = number of prey *i*/total number of prey × 100) and percentage by weight (%*W* = weight of prey *i*/total weight of all prey × 100). These values were used to calculate the prey‐specific index of relative importance (%PSIRI) (Brown et al. 2012) according to the equation:

$$\%\text{PSIRI}=\left[\%F\left(\%{\mathrm{PN}}_{i}+\%{\mathrm{PW}}_{i}\right)\right]/2$$

where:

%*F* is the percentage frequency of occurrence of a specific prey in all samples; %PN_*i*_ = $\frac{\sum_{j=1}^{n}N_{\mathrm{ij}}\%}{n_{i}}$ and %PW_*i*_ = $\frac{\sum_{j=1}^{n}W_{\mathrm{ij}}\%}{n_{i}}$ are the substitution of the IRI prey percent number %*N* and prey percent mass %*W* with their corresponding prey‐specific abundances.

Finally, the better‐preserved specimens were fixed in 70% ethanol solution and stored in the fish collection of the Ecology Laboratory of the University of Bari Aldo Moro (Italy).

## 3 Results

**FIGURE 3:**
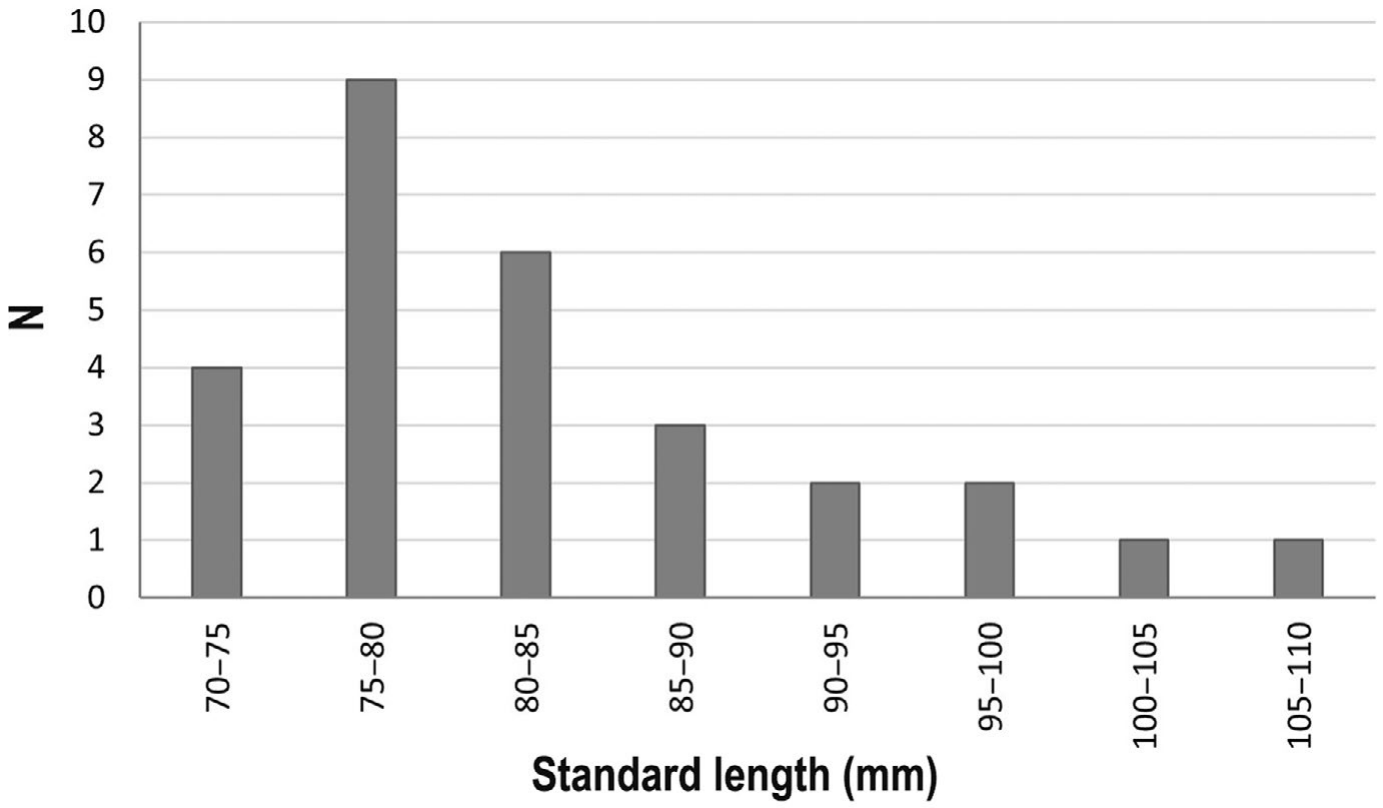
Length–frequency distribution of *L. gemellarii* sampled in the northern Ionian Sea during August–September 2023.

The range of morphometric measurements and meristic counts of *L. gemellarii* individuals is shown in Table 1. The sizes of the individuals were between 72 and 109 mm SL, even if most of them had SL between 75 and 80 mm, while a few exceeded the length of 90 mm SL (Figure 3).

**TABLE 1:** Range of morphometric measurements and meristic counts of the specimens of *L. gemellarii* sampled in the northern Ionian Sea.

Morphometric characters (mm)	Range
Standard length (SL)	72–109
Total weight (g)	65–217
Head length (HL)	23–32
Interorbital length	6–10
Preorbital length	4–8
Eye–operculum distance	9–16
Eye diameter	5–9
Upper jaw length	19–25
Lower jaw length	17–23
Operculum–tail distance	63–85
Predorsal length	33–43
Prepectoral length	25–35
Preanal length	51–67
Preventral length	38–51
Pectoral fin length	5–8
Pelvic fin length	10–16
Dorsal fin base	19–28
Anal fin base	13–18
Maximum trunk height after the operculum	17–22
Minimum trunk height at caudal peduncle	8–12
Maximum height at anus	15–23

Meristic characters	Count
Dorsal fin rays	17
Anal fin rays	14
Pectoral fin rays	12
Pelvic fin rays	10

**FIGURE 4:**
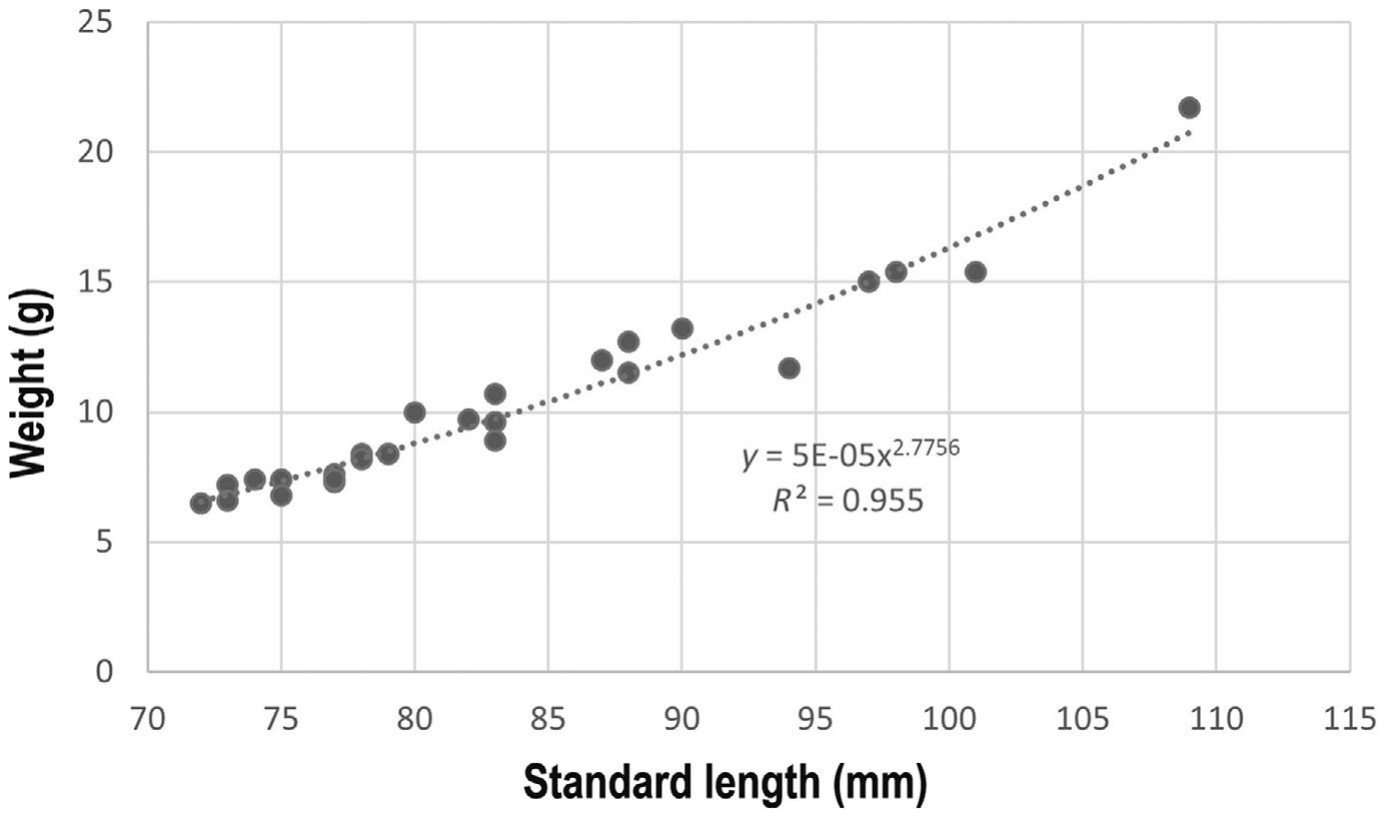
Length–weight relationship of *L. gemellarii* sampled in the northern Ionian Sea during August–September 2023.

Considering the growth in weight, the size–weight relationship was computed for the standard length (SL, in mm) and body weight (W, in g) (Figure 4). The function was as follows:

$$W=\text{5E}-05{\mathrm{SL}}^{2.78},r^{2}=0.96.$$

The presence of the supra‐caudal luminous gland (Figure 2) allowed the identification of 15 males in the sample; the absence of this structure in the other 13 individuals led to the assumption that they could be female specimens, although the infra‐caudal luminous gland was not always easily identifiable due to the imperfect preservation of the samples.

**FIGURE 5:**
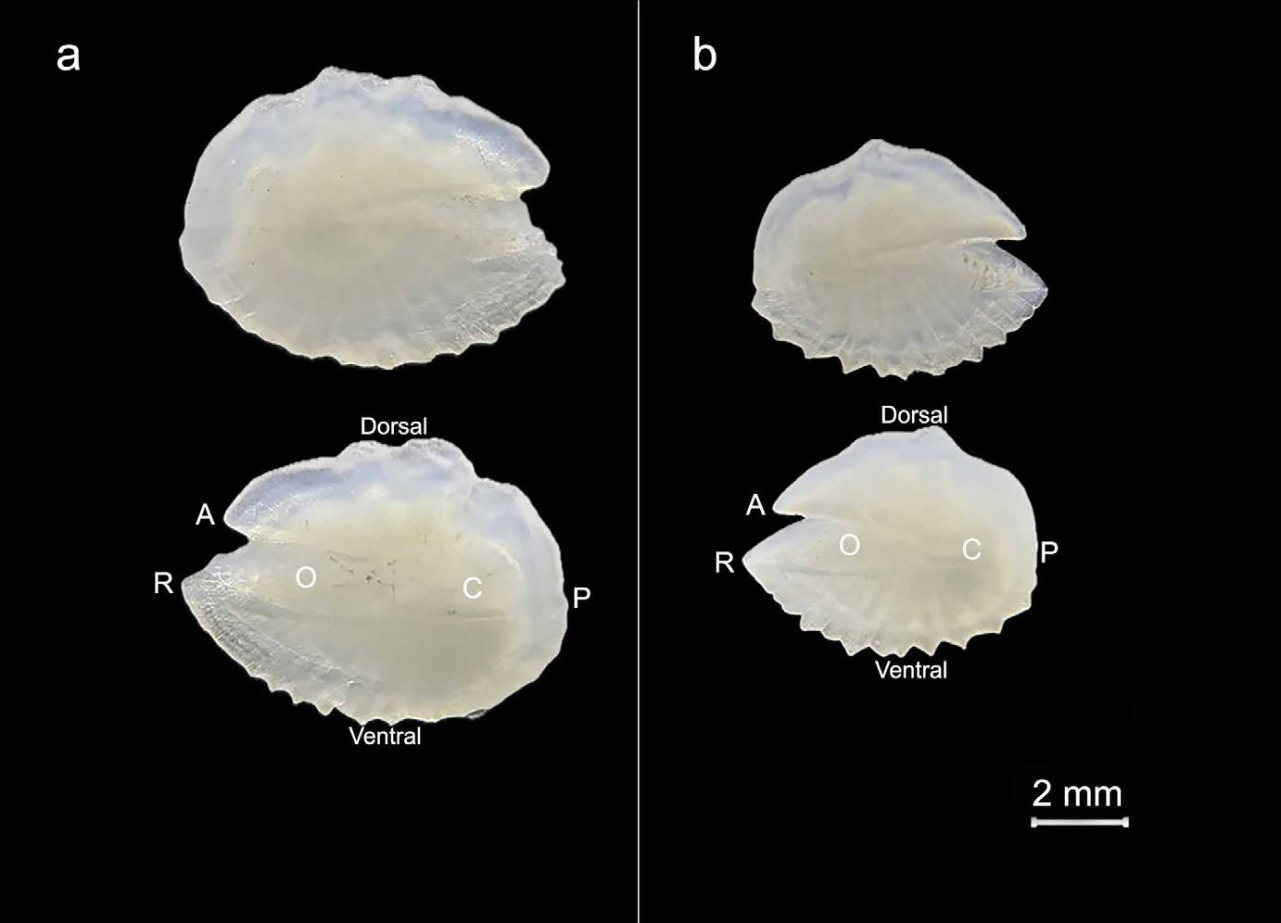
*L. gemellarii* otoliths: (a) SL = 109 mm; (b) SL = 72 mm. A, antirostrum; C, cauda; O, ostium; P, posterior; R, rostrum.

The identification of the species was also confirmed by the morphology of the otoliths, taken from all samples: each otolith was moderately elongated, with a postcentral *umbo* on the outer face, a narrow *ostium*, considerably longer than the *cauda*, an inner face only bent along the horizontal axis, a long *rostrum*, a well‐developed postdorsal angle, a depressed predorsal rim, denticles on the ventral rim and dorsal *rim* of the straight *ostium* (Figure 5).

**FIGURE 6:**
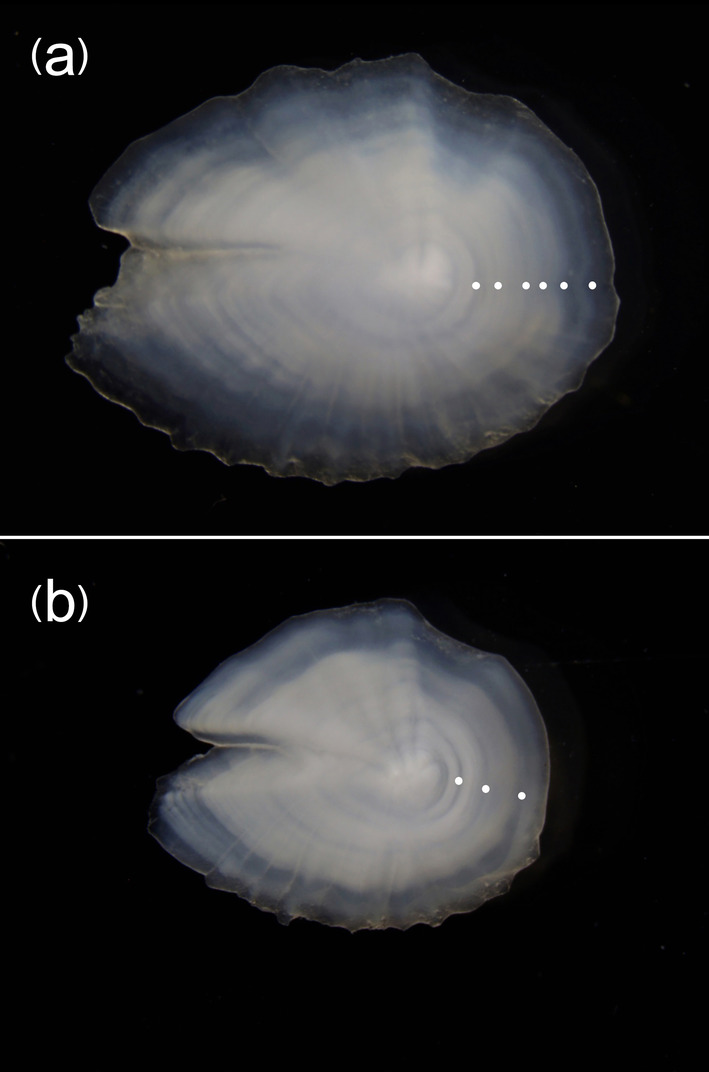
(a) Otolith of the largest individual of *L. gemellarii* (SL = 109 mm) with the presence of six growth rings; (b) otolith of individual of *L. gemellarii* (SL = 83 mm) with the presence of three growth rings.

**FIGURE 7:**
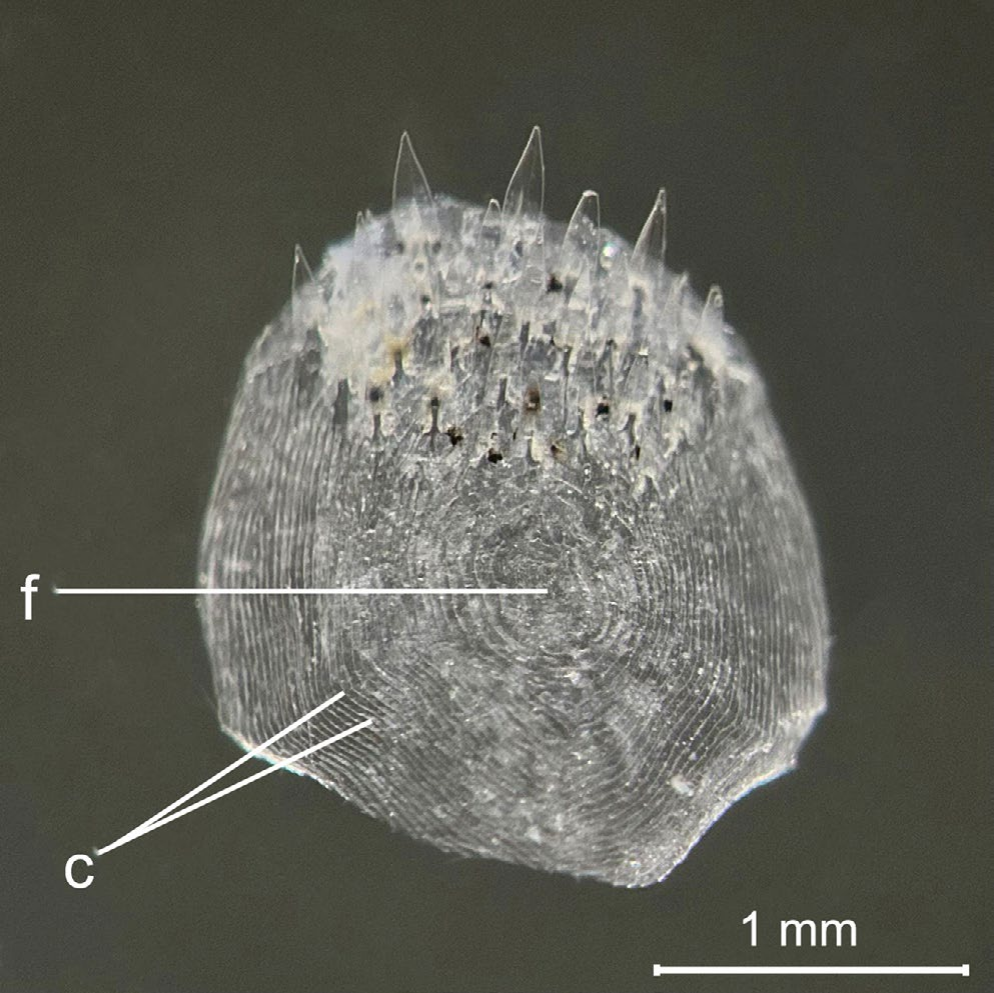
*L. gemellarii* cycloid scale. c, circuli; f, focus.

Otoliths analysis showed that the size range of 72–109 mm SL observed for the species in the study area corresponded to an age range between 2 and 6 years (Table 2). The majority of the sampled specimens belonged to the 2 year age‐class. Figure 6 shows the otolith of the largest individual (SL = 109 mm) with the alternance of six opaque and transparent growth rings, and that of specimen of SL = 83 mm with the alternance of three opaque and transparent growth rings. The scales, taken from the lateral line, were small, cycloid, and showed distinct, continuous, and concentric *circuli* around the *focus*, the anterior field with a smooth irregular margin, while the posterior field was rounded with a spinous margin (Figure 7).

**TABLE 2:** Age‐length key for *L. gemellarii* sampled in the northern Ionian Sea.

Standard length class (mm)	Age class (year)				
	2	3	4	5	6
70–75	6				
75–80	8				
80–85		5			
85–90		1	3		
90–95			1		
95–100				2	
100–105				1	
105–110					1
*N*	14	6	4	3	1

A high level of stomach fullness was recorded in all specimens, in fact, no empty stomachs were found. The prey items of the specimens of *L. gemellarii* and their occurrence are shown in Table 3. A total of 14 prey categories were identified in the stomach contents belonging to three major taxonomic groups (Mollusca, Crustacea, and Teleostea). In terms of %PSIRI most of the diet consisted of Crustacea (%PSIRI = 82.52), among which Crustacea n.d. (%PSIRI = 61.76) and Decapoda n.d. (%PSIRI = 10.90) were the most important, then Euphausiacea (%PSIRI = 3.29), Tanaidacea (%PSIRI = 1.25), and Amphipoda (%PSIRI = 0.37). The second most represented taxon was that of Teleostea (%PSIRI = 17.34), within which Osteichtyes n.d. (%PSIRI = 9.69) and the Myctophidae family (%PSIRI = 4.19) were the most represented. Mollusca was the prey of lesser importance with %PSIRI equal to 0.14.

**TABLE 3:** Diet composition of *L. gemellarii* sampled in the northern Ionian Sea.

Taxa	%*F*	%*N*	%*W*	PSIRI	%PSIRI
*Pasiphaea sivado*	3.57	0.76	10.41	194.95	1.95
*Squilla mantis*	3.57	0.76	1.37	175.55	1.76
Anomura nd	3.57	0.76	0.30	64.69	0.65
Brachyura nd	3.57	0.76	0.02	61.80	0.62
Decapoda nd	17.86	6.06	30.95	1089.84	10.90
Amphipoda nd	3.57	0.76	0.01	36.66	0.37
Euphausiacea nd	14.29	3.79	14.50	328.50	3.29
Tanaidacea nd	10.71	2.27	0.32	124.60	1.25
Crustacea nd	85.71	58.33	37.91	6175.66	61.76
Total Crustacea		74.24	95.77	8252.25	82.52
Cefalopoda nd	3.57	0.76	0.0001	13.77	0.14
Total Cephalopoda		0.76	0.0001	13.77	0.14
*Chauliodus sloani*	7.14	1.52	0.26	98.30	0.98
*Hygophum benoiti*	7.14	2.27	1.85	247.87	2.48
Myctophidae nd	7.14	5.30	1.19	419.26	4.19
Osteichtyes nd	42.86	15.91	0.92	968.55	9.69
Total Osteichtyes		25.00	4.23	1733.98	17.34

Except for the decapods *Squilla mantis* (%PSIRI = 1.76) and *Pasiphaea sivado* (%PSIRI = 1.95), the Sloane's viperfish *Chauliodus sloani* (%PSIRI = 0.98) and the Benoit's lanternfish *Hygophum benoiti* (%PSIRI = 2.48), the identification of prey items at species level was not possible due to the advanced status of the digestion process.

**FIGURE 8:**
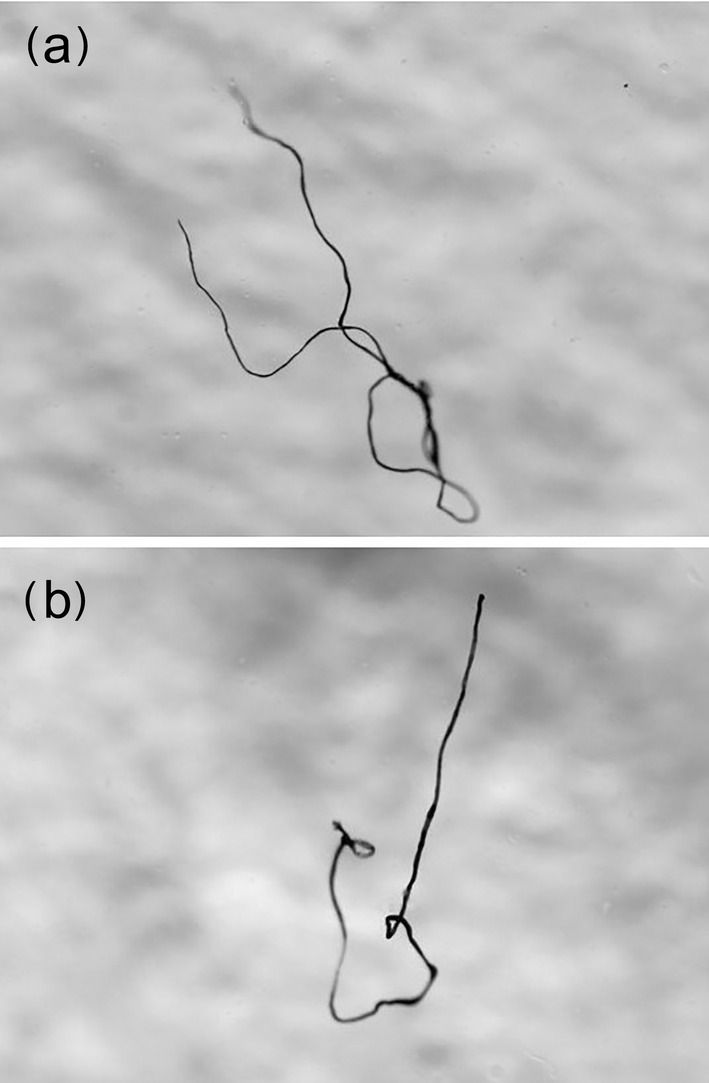
Plastic fibers found in the gut content of two individuals of *L. gemellarii* sampled in the northern Ionian Sea a = 40 mm; b = 32 mm.

Parasitic nematodes of the genus *Anisakis* and two pieces of plastic fiber measuring 4.0 and 3.2 mm in length (large microplastics) were found in the stomach contents of two samples (Figure 8).

## 4 Discussion

This paper reports the first ecological data of the mesopelagic fish *L. gemellarii* in the Central Mediterranean and their first records from the northern Ionian Sea. Previously, the species was incorrectly reported from the northern Ionian Sea (Maiorano et al. 2010; Capezzuto et al. 2010) due to its morphological similarity with the species of the same genus *L. dofleini* . The distinction between the two species of genus *Lobianchia* is based: (i) on the position of the three supra‐anal photophores, aligned in *L. gemellarii* and curved in the species *L. dofleini* and (ii) on the pre‐caudal photophores that in *L. gemellarii* are spaced regularly, unlike *L. dofleini* where Prc4 is located on the basis of the caudal fin rays, distant from the other three. Due to the significantly smaller size of the lanternfish samples previously found in the northern Ionian Sea, which were typical of the congeneric species, such samples were re‐examined. Comparison with recent records of *L. gemellarii* has allowed the identification of the previous error and the re‐classification of the old samples as *L. dofleini* .

*L. gemellarii* had been found in the Pacific (Hulley 1984; Kashkin 1989), Atlantic (Hulley 1981; Nafpaktitis 1968; Nafpaktitis et al. 1977), Indian Ocean (Hulley 1984) and in the Mediterranean Sea (Cihangir et al. 2003; Koukouras 2010; Battaglia et al. 2015; Borg et al. 2023), where the species has been recorded along the Sicilian coast of the Strait of Messina (Cocco 1838; Battaglia et al. 2015), near the shore of Malta (Borg et al. 2023), from the eastern Ionian Sea along the Greek coasts (Koukouras 2010) and from the Aegean Sea along the Turkish coasts (Cihangir et al. 2003). A few reports suggest that this species is rare in the Mediterranean Sea. To date, records of Cocco's lanternfish have only been reported from the central and eastern Mediterranean Sea. Considering that *L. gemellarii* has a tropical distribution pattern (Hulley 1992) and that temperature affects lantern fish distribution (Brandt 1983), we can assume that the expansion of its distribution may be due to the rise in temperature detected also in the Mediterranean and in the study area (Sion et al. 2024).

Concerning morphometric and meristic data, the present results agree with those of individuals of similar length from the Aegean Sea (Cihangir et al. 2003) and with data reported for this species by Nafpaktitis (1968) and Hulley (1984), except for the maximum recorded length of 60 mm, because in this study the standard length of all individuals was greater, up to a maximum of 109 mm, which is the largest size ever found for the species. However, Nafpaktitis et al. (1977) and Hulley (1984) report that specimens of *L. gemellarii* up to a size of 100 mm may be occasionally found outside spawning areas, and these expatriate specimens often lack luminous caudal glands. In this study, the supra‐caudal luminous gland was present in most of them. The arrangement of the photophores also agreed with the description given by Sutton et al. (2020), although sometimes they were not all visible due to damage caused during the capture.

Although the sampled specimens, being quite large and with most of the photophores visible, allowed for an unequivocal species identification, molecular confirmation could still be useful.

The slope of the size–weight relationship suggests the existence of a negative allometry in the growth of *L. gemellarii* , indicating a relatively slower growth in weight than in length across the development. This negative allometry is in agreement with that observed in the Strait of Messina and the Strait of Sicily (Battaglia et al. 2015), while it disagrees with that reported in the northwestern Mediterranean, where significantly positive allometric relationships were observed for several myctophid species, for example, *Benthosema glaciale* , *Ceratoscopelus maderensis* , *Diaphus holti* , *Lampanyctus crocodilus* , *L. pusillus* , *Notoscopelus elongatus* , *Symbolophorus veranyi* , and the congeneric *Lobianchia dofleini* (Olivar, Moli, and Bernal 2013). However, this positive allometry reported in several myctophids was in contrast to data published by Battaglia et al. (2010).

The otolith morphology also agrees with the information provided by Smale, Watson, and Hecht (1995), Schwarzhans (2013), and Lin et al. (2017). The analysis of growth rings with an estimated age is shown for the first time for the species in the present study.

The morphology of the scales is described for the first time and has not been compared to other species of the genus *Lobianchia*.

As regards, the stomach content of *L. gemellarii* , this is the first detailed information reported on the feeding of the adult of the species. Previously, Conley and Hopkins (2004) examined the feeding ecology of two larvae of *L. gemellarii* in which soft‐bodied thaliacean and larvacean prey dominated and other common diet items included copepod eggs and protists. However, the feeding habits of adults were characterized by crustacean, decapod and teleost preys, and other common diet items included small crustaceans such as amphipods, euphausiaceans, and tanaidaceans. Increasing the size of the specimens increases the size range of available prey. The presence in the diet of pelagic preys such as *P. sivado* or mesopelagic species like *H. benoiti* and *C. sloani* , as well as benthic preys like *S. mantis* , highlights the migratory behavior of the species. *H. benoiti* and, in general, the family Myctophidae are quite common prey items in the diet of other myctophids (Bernal et al. 2015). Additionally, the predator *C. sloani* has been found in the stomachs of *Diaphus metapoclampus* (Battaglia et al. 2014) and, in general, Stomiiformes have been found in *Electrona risso* as well (Battaglia et al. 2016). In the congeneric species *L. dofleini* , in the Northwest Atlantic, copepods were the most represented prey in the diet, whereas in the stomachs of *L. gemellarii* , no copepods were found. It is important to increasingly understand the prey items of these mesopelagic fish because, in addition to better explaining their significant role as biological conveyors, they could prove useful for understanding the biodiversity of the deep marine environment (Capezzuto et al. 2022; Capezzuto et al. 2024). However, the knowledge on the ecology and distribution of these species has progressed more in the open ocean than in the Mediterranean Sea (Battaglia et al. 2015).

The finding of plastic fibers in two stomachs confirms what has already been observed in other areas of the Mediterranean and the Atlantic in other species of myctophids (Romeo et al. 2016; Ferreira et al. 2023; Laface et al. 2023). The ingestion of plastic may represent a risk for vertical migrant lanternfishes due to the increase in buoyancy (Romeo et al. 2016). Despite the low ingestion rate of microplastics by mesopelagic fish, the quantities of mesopelagic specimens consumed by predators imply that the transfer of microplastics across trophic levels may reach significant magnitudes at higher levels (Laface et al. 2023).

The current research sheds light on the preliminary ecological data of the mesopelagic fish *L. gemellarii* in the central Mediterranean. Despite the additional knowledge provided, the scant abundance of the species has not allowed further analysis of its life cycle. Further sampling will help bridge the gap in the ecology of *L. gemellarii* , which is so important due to its role as a keystone species in the energy flow in the deep marine environment.

## Author Contributions

**Cristina Turco:** data curation (lead), formal analysis (equal), investigation (equal), methodology (equal), writing – original draft (equal). **Letizia Sion:** formal analysis (equal), methodology (equal), validation (equal). **Gabriele Galasso:** data curation (equal), formal analysis (equal). **Gianfranco D'Onghia:** supervision (lead). **Francesca Capezzuto:** conceptualization (lead), data curation (equal), formal analysis (equal), investigation (equal), methodology (lead), writing – original draft (lead), writing – review and editing (lead).

## Ethics Statement

All specimens analyzed in this study were collected from the fishery (Data Collection Framework [DCF]; EU Reg. 199/2008). Therefore, this study does not comply with the European Commission recommendations (Directive 2010/63/EU of the European Parliament and of the Council of 22 September 2010) or with Italian National Law (Decree Law n. 26 of 4 March 2014) on the protection of animals used for scientific experiment. All authors have read, understood, and have complied as applicable with the statement on “Ethical responsibilities of Authors” as found in the Instructions for Authors and are aware that with minor exceptions, no changes can be made to authorship once the paper is submitted.

## Conflicts of Interest

The authors declare no conflicts of interest.

## Data Availability

*Lobianchia gemellarii* dataset from MEDITS (doi: 10.5061/dryad.612jm64cw) is in Dryad. Reviewer URL: https://datadryad.org/stash/share/g02Zeb7tFUVBkk29oFySm9JmXUt0gwcjjighkLi2qp8.
